# Laser Beam Pointing Stabilization Control through Disturbance Classification

**DOI:** 10.3390/s21061946

**Published:** 2021-03-10

**Authors:** Hui Chang, Wen-Qi Ge, Hao-Cheng Wang, Hong Yuan, Zhong-Wei Fan

**Affiliations:** 1 Aerospace Information Research Institute, Chinese Academy of Sciences, Beijing 100094, China; changhui@aircas.ac.cn (H.C.); wanghaocheng@aoe.ac.cn (H.-C.W.); yuanh@aoe.ac.cn (H.Y.); 2 School of Electronic, Electrical and Communication Engineering, University of Chinese Academy of Sciences, Beijing 100049, China; 3 University of Chinese Academy of Sciences, Beijing 100049, China

**Keywords:** recurrent neural network, disturbance classification, beam pointing stabilization, Jacobian matrix, position sensitive devices

## Abstract

In laser systems, beam pointing usually drifts as a consequence of various disturbances, e.g., inherent drift, airflow, transmission medium variation, mechanical vibration, and elastic deformation. In this paper, we develop a laser beam pointing control system with Fast Steering Mirrors (FSMs) and Position Sensitive Devices (PSDs), which is capable of stabilizing both the position and angle of a laser beam. Specifically, using the ABCD matrix, we analyze the kinematic model governing the relationship between the rotation angles of two FSMs and the four degree-of-freedom (DOF) beam vector. Then, we design a Jacobian matrix feedback controller, which can be conveniently calibrated. Since disturbances vary significantly in terms of inconsistent physical characteristics and temporal patterns, great challenges are imposed to control strategies. In order to improve beam pointing control performance under a variety of disturbances, we propose a data-driven disturbance classification method by using a Recurrent Neural Network (RNN). The trained RNN model can classify the disturbance type in real time, and the corresponding type can be subsequently used to select suitable control parameters. This approach can realize the universality of the beam stabilization pointing system under various disturbances. Experiments on beam pointing control under several typical external disturbances are carried out to verify the effectiveness of the proposed control system.

## 1. Introduction

Lasers have been used in many important fields such as laser processing, laser ranging, and laser communication. The stability of beam pointing is a key feature that impacts manufacturing precision, location accuracy, and communication efficiency. In practice, the drift of a laser beam away from a setting direction could be caused by many disturbance sources, including: (1) inherent drift, which is related to the temperature distribution and micro motion in the laser cavity; (2) air disturbance, such as airflow; (3) transmission medium variation; (4) mechanical vibration, arising from the motion of electric motors and coolers; (5) elastic deformation, which is caused by forces on mechanical components.

Thus, these factors should be mitigated in a beam pointing stabilization control system with steering mirrors and position sensing detectors. A beam can be defined by a four-dimensional vector: positions and angles along two orthogonal directions. Two Fast Steering Mirrors (FSMs) are adopted to stabilize the beam, and two Position Sensitive Devices (PSDs) are used to detect the positions and angles. In [1], the beam stabilization system adopts the matrix decoupling technology, which needs an accurate beam delivery mathematic model. The parameters of the beam delivery mathematic model are achieved by the detailed parameters of the lens and optical path. Wang et al. proposed a beam correction principle by calibrating the beam pointing path and beam position path [2]. Then, the relationship between the rotation angle and the signal of the PSDs can be used to correct the beam drift. In [3], the tracking control of beam pointing was built on the Jacobian matrix, which can be efficiently self-calibrated by active motion. However, we find that its control performance relies on the parameter setting, and the optimal parameter is highly related to the disturbance’s feature. As mentioned above, there are many types of disturbances, and thus, a controller with constant parameters can hardly perform well when encountering various external disturbances. Therefore, it is desired to select proper control parameters according to the current disturbance type.

In a beam pointing control system, it is necessary to analyze the characteristics of disturbances and classify disturbance types in order to select suitable control parameters. In recent years, deep learning technologies [4] have been applied to many fields, such as objection recognition [5], speech recognition [6], and natural language processing [7], due to their powerful discriminative ability and data-driven manner. Specifically, in this paper, we consider disturbance classification of the beam pointing stabilization control system using a Recurrent Neural Network (RNN) [8], which shows effective performance in time series tasks [9]. However, the basic RNN suffers from vanishing and exploding gradient problems for long-term dependency tasks [10]. To avoid these limitations, Long Short-Term Memory (LSTM) [11] and the Grated Recurrent Unit (GRU) [12] have been proposed based on grating mechanisms. Beam pointing drift can be regarded as a multi-dimensional temporal signal. However, to the best of our knowledge, the existing works seldom leverage deep neural networks on the disturbance classification task for laser systems.

In this paper, a disturbance classification method based on LSTM and the GRU is proposed to select suitable control parameters for the beam pointing stabilization control system. The key advantages are as follows: the classification model is learned in a data-driven manner without relying on artificial analysis and tuning; the deep learning approach provides good generalization ability; the disturbance classification can realize the universality of the system under various disturbances. In our experiment, we first analyze the kinematic model of the beam pointing stabilization control system based on the beam delivery matrix. In addition, we design a feedback controller based on the Jacobian matrix [3]. The Jacobian matrix is adaptive to the changes on the beam path by self-calibrating and can be calibrated efficiently with active motion and linear least squares optimization. Then, we capture the beam pointing drift data under several types of disturbances and classify disturbances using LSTM/the GRU. Finally, according to the result of the disturbance classification, suitable parameters of the control system can be selected.

## 2. Control System Description

**Figure 1:**
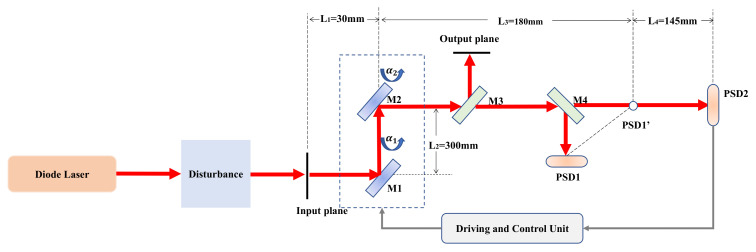
Schematic of the beam pointing control system. M1, Mirror 1; PSD, Position Sensitive Device.

A diode laser, with a 650 nm center wavelength and a 5 mW output power, was used as the light source. The diameter of the laser beam is 2 mm. As shown in Figure 1, M1 and M2 are the two highly reflective FSMs. The size of the FSM is 60 × 60 mm. Each mirror was mounted with two micro-stepping motors to turn the reflective angles in both horizontal and vertical directions independently. M3 is a partial reflective mirror. A small part of the laser was transmitted into the PSD1 and PSD2 for positioning. PSD1 and PSD2 are position sensitive detectors (GmbH. TEM Messtechnik) with a sub-micro-rad and a 350 nm to 1100 nm spectral range. The chip sizes of PSD1 and PSD2 are 9×9 mm and 4×4 mm, respectively. M4 is a beam splitter mirror. The optical distance from M3 to PSD1 is shorter than that from M3 to PSD2. Each PSD measures the x-y position of the laser spot on it. The output beam pointing can be uniquely determined by the two laser spots’ positions on the PSDs. The driving and control unit acquires data from the PSDs and generates control signals for the micro-stepper to regulate the beam pointing. Some key parameters are defined as follows: $L_{1}$ is the optical distance between the input plane and M1; $L_{2}$ is the optical distance between M1 and M2; $L_{3}$ is the optical distance from M2 to PSD1’. PSD1’ is the image of PSD1 to M4, and $L_{4}$ is the optical distance from PSD1’ to PSD2, shown as Figure 1. $\alpha_{1}$ and $\alpha_{2}$ are the steering angles of M1 and M2, respectively.

## 3. Modeling Methods

### 3.1. Optical Model

Each beam has two orthogonal directions. In this section, the horizontal direction is analyzed, which can be easily extended to the vertical direction. In general, a laser beam can be defined by two coordinates: the position *r* and the angle θ from the optical axis, namely 2 degrees-of-freedom (DOFs). $r_{in}$ and $\theta_{in}$ are the position and angle upon entering the optical path. $r_{out}$ and $\theta_{out}$ are the position and angle after propagating by a non-negative distance *z* [13]. Because the displacements and angles are assumed small, we have the approximate relations,

(1) $$\left(\begin{array}{c} r_{out}\\ \theta_{out} \end{array}\right)=\left(\begin{array}{cc} 1 & z\\ 0 & 1 \end{array}\right)\left(\begin{array}{c} r_{in}\\ \theta_{in} \end{array}\right)$$

Meanwhile, let $r_{0}$ and $\theta_{0}$ be the input beam parameters of the beam pointing control system. According to (1), $r_{1}$ and $\theta_{1}$ on PSD1’ can be calculated by the two laser spots’ positions on the PSDs,

(2) $$\left(\begin{array}{c} r_{1}\\ \theta_{1} \end{array}\right)=\left(\begin{array}{cc} 1 & L_{1}+L_{2}+L_{3}\\ 0 & 1 \end{array}\right)\left(\begin{array}{c} r_{0}\\ \theta_{0} \end{array}\right)+2\left(\begin{array}{cc} L_{2}+L_{3} & L_{3}\\ 1 & 1 \end{array}\right)\left(\begin{array}{c} \alpha_{1}\\ \alpha_{2} \end{array}\right)$$

Since $\theta_{1}$ cannot be achieved by PSD1 directly, it can be calculated by,

(3) $$\theta_{1}=\left(r_{1}-r_{2}\right)/L_{4}$$

Because $L_{4}$ is constant, we substitute (3) into (2),

(4) $$\left(\begin{array}{c} r_{1}\\ r_{2}-r_{1} \end{array}\right)=\left(\begin{array}{cc} 1 & L_{1}+L_{2}+L_{3}\\ 0 & L_{4} \end{array}\right)\left(\begin{array}{c} r_{0}\\ \theta_{0} \end{array}\right)+2\left(\begin{array}{cc} L_{2}+L_{3} & L_{3}\\ L_{4} & L_{4} \end{array}\right)\left(\begin{array}{c} \alpha_{1}\\ \alpha_{2} \end{array}\right)$$

When $\alpha_{1}=\alpha_{2}=0$, a beam offset vector is defined by:

(5) $$\left(\begin{array}{c} r_{10}\\ r_{20}-r_{10} \end{array}\right)=\left(\begin{array}{cc} 1 & L_{1}+L_{2}+L_{3}\\ 0 & L_{4} \end{array}\right)\left(\begin{array}{c} r_{0}\\ \theta_{0} \end{array}\right)$$

The kinematic model can be deduced by substituting (5) into (4):

(6) $$\left(\begin{array}{c} r_{1}\\ r_{2}-r_{1} \end{array}\right)=\left(\begin{array}{c} r_{10}\\ r_{20}-r_{10} \end{array}\right)+2\left(\begin{array}{cc} L_{2}+L_{3} & L_{3}\\ L_{4} & L_{4} \end{array}\right)\left(\begin{array}{c} \alpha_{1}\\ \alpha_{2} \end{array}\right)$$

Due to the beam delivery matrix, we have a linear kinematic model. Once the input beam pointing drifts, the beam pointing control system should regulate both the position $r_{1}$ and angle $\left(r_{2}-r_{1}\right)$ of the laser beam by setting steering angles $\alpha_{1}$ and $\alpha_{2}$.

### 3.2. Kinematic Model

According to (6), the kinematic model with a 4-DOF pose of the beam can be given by the local linear approximation:

(7) $$\left[\begin{array}{c} r_{1}\\ r_{3}\\ r_{2}-r_{1}\\ r_{4}-r_{3} \end{array}\right]=\left[\begin{array}{c} r_{10}\\ r_{30}\\ r_{20}-r_{10}\\ r_{40}-r_{30} \end{array}\right]+\underset{J}{\underset{︸}{\left[\begin{array}{cccc} J_{11} & J_{12} & J_{13} & J_{14}\\ J_{21} & J_{22} & J_{23} & J_{24}\\ J_{31} & J_{32} & J_{33} & J_{34}\\ J_{41} & J_{42} & J_{43} & J_{44} \end{array}\right]}}\left[\begin{array}{c} \alpha_{1}\\ \alpha_{2}\\ \alpha_{3}\\ \alpha_{4} \end{array}\right]$$

where $r_{1}$ and $r_{3}$ are the 2D positions on PSD1, which represent the beam’s horizontal and vertical position. $r_{2}$ and $r_{4}$ are the 2D positions on PSD2. Meanwhile, $\left(r_{2}-r_{1}\right)$ and $\left(r_{4}-r_{3}\right)$ represent the beam’s horizontal and vertical angle. $\alpha_{1}$ and $\alpha_{3}$ are the steering angles of M1. $\alpha_{2}$ and $\alpha_{4}$ are the steering angles of M2. J is the Jacobian matrix. Thus, the four actual steering angles coordinately change the laser spots’ positions on the two PSDs by the Jacobian matrix.

### 3.3. Active Motion Based Calibration

To build the control system, we re-formulate the kinematic model in (7) using the feedback vector $x\in R^{4\times 1}$ and the step-motor order $u\in R^{4\times 1}$,

(8) $$\underset{x}{\underset{︸}{\left[\begin{array}{c} x_{1}\\ x_{2}\\ x_{3}\\ x_{4} \end{array}\right]}}=\underset{x_{0}}{\underset{︸}{\left[\begin{array}{c} x_{10}\\ x_{20}\\ x_{30}\\ x_{40} \end{array}\right]}}+\underset{J}{\underset{︸}{\left[\begin{array}{cccc} J_{11} & J_{12} & J_{13} & J_{14}\\ J_{21} & J_{22} & J_{23} & J_{24}\\ J_{31} & J_{32} & J_{33} & J_{34}\\ J_{41} & J_{42} & J_{43} & J_{44} \end{array}\right]}}\underset{u}{\underset{︸}{\left[\begin{array}{c} u_{1}\\ u_{2}\\ u_{3}\\ u_{4} \end{array}\right]}}$$

where $x_{1}=r_{1}$, $x_{2}=r_{3}$, $x_{3}=r_{2}-r_{1}$, $x_{4}=r_{4}-r_{3}$. $u_{i}$ is the discrete order of the *i*th axis of the two FSMs, instead of the actual steering angle.

In order to calibrate the kinematic model in a short time, a time series of predefined orders is sent to the step-motors to generate active motion; meanwhile, a series of the laser spot positions on the two PSDs is sampled. The length of the time series is *n*. Thus, we have:

(9) $$\underset{P}{\underset{︸}{\left[\begin{array}{ccccc} x_{11} & x_{12} & x_{13} & \cdots & x_{1n}\\ x_{21} & x_{22} & x_{23} & \cdots & x_{2n}\\ x_{31} & x_{32} & x_{33} & \cdots & x_{3n}\\ x_{41} & x_{42} & x_{43} & \cdots & x_{4n} \end{array}\right]}}=\underset{A}{\underset{︸}{\left[\begin{array}{ccccc} J_{11} & J_{12} & J_{13} & J_{14} & x_{10}\\ J_{21} & J_{22} & J_{23} & J_{24} & x_{20}\\ J_{31} & J_{32} & J_{33} & J_{34} & x_{30}\\ J_{41} & J_{42} & J_{43} & J_{44} & x_{40} \end{array}\right]}}\underset{U}{\underset{︸}{\left[\begin{array}{ccccc} u_{11} & u_{12} & u_{13} & \cdots & u_{1n}\\ u_{21} & u_{22} & u_{23} & \cdots & u_{2n}\\ u_{31} & u_{32} & u_{33} & \cdots & u_{3n}\\ u_{41} & u_{42} & u_{43} & \cdots & u_{4n}\\ 1 & 1 & 1 & \cdots & 1 \end{array}\right]}}$$

Using linear least squares optimization, the explicit solution to (9) is:

(10) $$A=PU^{T}{\left(UU^{T}\right)}^{-1}$$

Then, the Jacobian matrix J can be extracted from A. Thus, given an unknown or changed optical path, the kinematic model can be self-calibrated in a few seconds, making the control system adaptive to different optical paths and free of manual parameter tuning. As the Jacobian matrix can be conveniently calibrated, the static drift error of the optical model can be corrected.

### 3.4. Control Method

**Figure 2:**
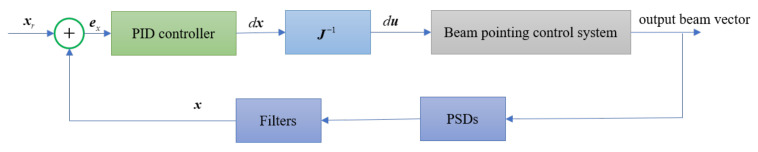
Block diagram of the beam pointing control system.

The control system is designed to reduce drifts caused by the mentioned disturbances, as shown in Figure 2. The Proportional Integral Differential (PID) feedback controller is adopted as the control law. The raw signals sensed by the PSDs are processed by a low-pass filter. The controller compares the 4D reference beam vector $x_{r}\in R^{4\times 1}$ and the feedback beam vector $x\in R^{4\times 1}$. Then, the PID controller is used to calculate the incremental movement of two PSD positions,

(11) $$dx\left(t\right)=k_{P}e_{x}\left(t\right)+k_{I}\sum_{i=0}^{t}e_{x}\left(i\right)+k_{D}\left(e_{x}\left(t\right)-e_{x}\left(t-1\right)\right)$$

where $k_{P}$, $k_{I}$, and $k_{D}$ are the proportional, integral, and differential gains, respectively. Finally, the incremental movement of two PSD positions dx is transformed to the incremental order du of the two FSMs, using the inverse of the Jacobian matrix, namely,

(12) $$du=J^{-1}dx$$

The increment order is sent to the FSM controller, so that the actual steering angles of the two FSMs are adjusted to reduce the error $e_{x}$.

## 4. Disturbance Source Classification Method

**Figure 3:**
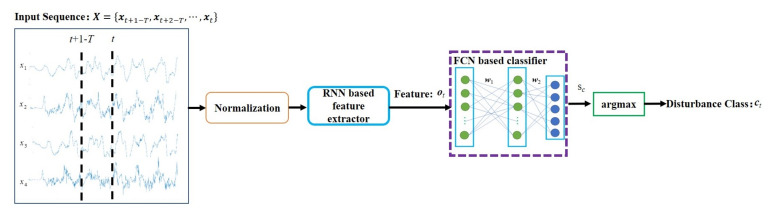
Pipeline of deep learning based disturbance classification.

In this paper, we assume the five typical disturbances for a pulsed laser system, including the inherent drift, air steam, transmission medium variation, mechanical motion, and elastic deformation. The deep learning methods are used for classification according to the temporal patterns of these disturbances. The pipeline of the proposed method is depicted in Figure 3.

At time step t, the current feedback beam vector and its historic states are combined as the input time series data $X=\left\{x_{t+1-T},x_{t+2-T},\cdots ,x_{t}\right\}\in \;R^{4\times T}$. *T* is the length of the time series. The raw data X are firstly normalized so that the mean and standard deviation of $\left\{x_{t+1-T},x_{t+2-T},\cdots ,x_{t}\right\}$ are zero and one, respectively, to minimize the influence on the amplitude variation and keep the relative temporal pattern. The normalized X is fed to the deep neural network to extract high-level features o, which can be realized based on the RNN. Then, the multi-dimensional feature vector o is fed to a two-layer fully connected network (FCN), and the output is the 5-dimensional activation vector $s=\left\{s_{1},s_{2},\cdots ,s_{5}\right\}$, as given by,

(13) $$s=w_{2}\cdot \left[\tanh(w_{1}\cdot o_{t}+b_{1})\right]+b_{2}$$

where $w_{i}$ and $b_{i}$ (i=1,2) are the weights and biases of the two layers of the FCN.

Then, the index c corresponding to the maximum activation value $s_{c}$ (c=1,2,⋯,5) is regarded as the identified disturbance class, namely,

(14) $$c=\underset{c}{\arg\max}\left(s_{c}\right)$$

In this work, both LSTM and the GRU are studied for feature extraction, as introduced in the following subsections.

### RNN Based Feature Extraction

**Figure 4:**
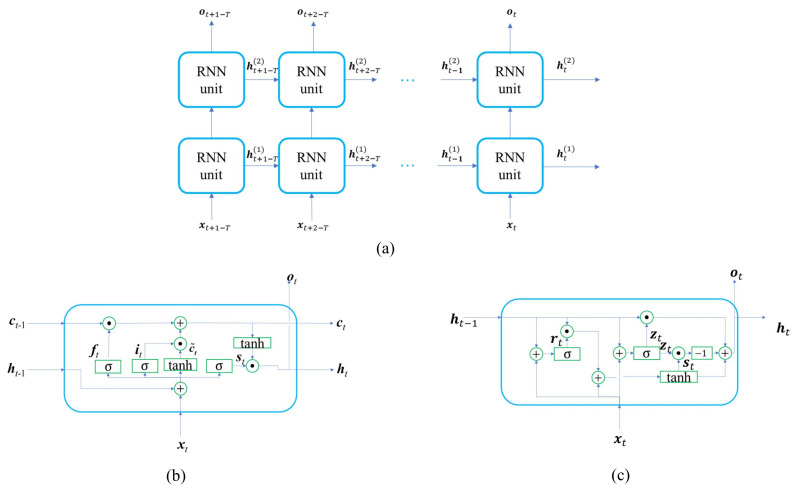
Architecture of the two-layer recurrent neural network. (**a**) Two-layer RNN, whose RNN unit can be realized by LSTM or the Grated Recurrent Unit (GRU). (**b**) LSTM unit. (**c**) GRU.

The Recurrent Neural Network (RNN) is preferred to handle the sequence input. It processes an input sequence in a recurrent manner. The samples in the time series are input to the RNN sequentially, and each time, the RNN unit receives both the new sample and the historical state of the last time step. Finally, the whole time series is processed, and the feature is propagated over time. A two-layer RNN is illustrated in Figure 4a. $x_{t}$ and $o_{t}$ respectively represent the input vector and output vector at time *t*. ${h_{t}}^{\left(n\right)}$ represents the hidden state of the RNN unit in the layer *n* (n=1,2) at time *t*. The standard RNN faces the problems of vanishing or exploding gradients during training. Therefore, we adopt the advanced RNN variants, namely LSTM and GRU.

The LSTM unit [11] solves the long-term dependency problem with three gates: forget gate, input gate, and output gate, as shown in Figure 4b. The three gates mainly determine the status of the unit by the following equations.

(15) $$f_{t}=\sigma \left(w_{f}\cdot \left[h_{t-1},x_{t}\right]+b_{f}\right)$$

(16) $$i_{t}=\sigma \left(w_{i}\cdot \left[h_{t-1},x_{t}\right]+b_{i}\right)$$

(17) $${\tilde{c}}_{t}=\tanh_{(}w_{c}\cdot \left[h_{t-1},x_{t}\right]+b_{c})$$

(18) $$c_{t}=f_{t}*c_{t-1}+i_{t}*{\tilde{c}}_{t}$$

(19) $$s_{t}=\sigma \left(w_{s}\cdot \left[h_{t-1},x_{t}\right]+b_{s}\right)$$

(20) $$h_{t}=s_{t}*\tanh\left(c_{t}\right)$$

where $f_{t}$, $i_{t}$, and $s_{t}$ represent the features of the forget gate, input gate, and output gate, respectively. ${\tilde{c}}_{t}$ is the candidate feature. $c_{t}$ and $h_{t}$ are the cell unit state and hidden feature propagated temporally over the RNN units. w and b are the weights and biases.

The GRU [12] is composed of a reset gate and update gate, as shown in Figure 4c. The computation of the GRU is given by,

(21) $$z_{t}=\sigma \left(w_{z}\cdot \left[h_{t-1},x_{t}\right]+b_{z}\right)$$

(22) $$r_{t}=\sigma \left(w_{z}\cdot \left[h_{t-1},x_{t}\right]+b_{r}\right)$$

(23) $$s_{t}=\tanh\left(w_{s}\cdot \left[h_{t-1}*r_{t},x_{t}\right]+b_{s}\right)$$

(24) $$h_{t}=\left(1-z_{t}\right)*h_{t-1}+z_{t}*s_{t}$$

where $x_{t}$, $h_{t}$, $z_{t}$, and $r_{t}$ respectively represent the input vector, output vector, update gate vector, and reset gate state. w and b are the weights and biases.

## 5. Experimental Results and Discussions

### 5.1. Kinematic Model Calibration Based on Active Motion

**Figure 5:**
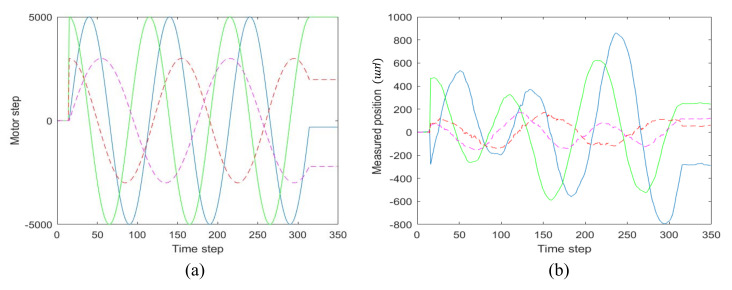
Active motion based data collection for kinematic calibration. (**a**) Motor steps by active motions. (**b**) Measured positions by active motions.

The four sine signals with different frequencies and amplitude, as shown in Figure 5a, are sent to the four step-motors of the FSMs, respectively. Thus, the four step-motors change the beam pointing actively. The resulting laser positions on the PSDs are measured and recorded as shown in Figure 5b. Using the least squares method, the kinematic model is obtained as:

(25) $$J=\left[\begin{array}{cccc} -0.0752637 & 0.00641021 & 0.105804 & 0.0092901\\ 0.0162511 & 0.0539132 & -0.0312554 & 0.0939795\\ -0.0271299 & -0.00583653 & -0.0244506 & -0.00735259\\ -0.0106072 & 0.0293533 & 0.0144381 & -0.0282633 \end{array}\right]$$

Root mean squared error (RMSE) between the actual beam vector observed by the PSDs and the beam vector estimated by the Jacobian matrix is calculated to evaluate the calibration accuracy.

(26) $$RMSE=\sqrt{\frac{\sum_{t=1}^{T}{\left({\hat{x}}_{t}-x_{t}\right)}^{2}}{T}}$$

where ${\hat{x}}_{t}$ and $x_{t}$ respectively represent the measured groundtruth beam vector and estimated output beam vector at time *t*. *T* represents the entire training sample time. The four values of the RMSE are 11.502,21.198,4.691, and 6.279. Therefore, the proposed calibration method provides an adequate accuracy and can be finished in just a few seconds.

### 5.2. Disturbance Type Classification

#### 5.2.1. Data Collection

**Figure 6:**
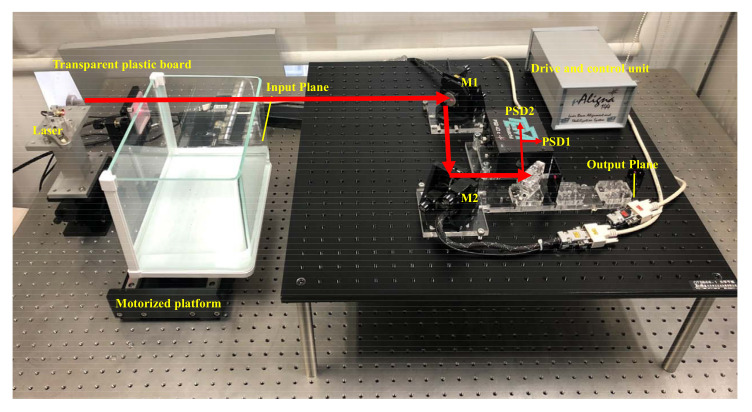
The photograph of the experimental setup.

In the disturbance classification experiments, the five types of disturbances were used, which include inherent drift, air disturbance, transmission medium variation, mechanical motion, and elastic deformation. Experiments were conducted in a laboratory at 30% humidity and 22 ${}^{\circ }$C. The disturbance was generated using a transparent box in the laser optical path, as shown in Figure 6. (1) The inherent drift produced by the laser itself was simulated without any external disturbances. (2) The bottom of the box was filled with hot water containing a constant-temperature heater, so that air disturbance in the glass box was generated. (3) A transparent plastic board with uneven thickness was mounted on the side wall of the box, and the box was moved randomly by a motorized platform, so that the transmission medium’s thickness was disturbed and the transmission medium variation produced. (4) The transparent box was moved by a motorized platform periodically, and mechanical motion disturbance was produced. (5) The transparent box was pressed by a motorized structure with a slightly varying period and amplitude, so elastic deformation was generated. The sampling frequency was 50 Hz. Thus, the five time series were collected, whose lengths were 8099, 8246, 8091, 8482, and 8219, respectively. The samples $X_{t}$ were obtained from the raw time series with a time step interval of 10. For each time series, the first 70% of the samples were used for training the model, and the remaining 30% of the samples were used for testing classification accuracy. Finally, two-thousand eight-hundred ten training samples and 1206 testing samples were obtained and normalized.

#### 5.2.2. Model Training

The aforementioned GRU based and LSTM based models were trained to classify the disturbance type. The training loss function was the standard multi-class cross-entropy loss. The training optimizer was the Adam optimizer [14]. The learning rate, epoch, and batch size were 0.001, 100, and 32, respectively. An Intel i7-8700K CPU and an NVIDIA RTX2080ti GPU were used as the deep learning environment. The disturbance classification model was implemented with the Pytorch library. Because the model’s performance was related to the number of neurons in the RNN, more neurons provided better discriminative ability, but led to a longer computation. To investigate the influence of different neuron numbers, we trained the deep learning models with different settings. The hidden neuron numbers of the GRU and LSTM were set to 64, 128, 256, and 512, respectively.

#### 5.2.3. Classification Evaluation

**Figure 7:**
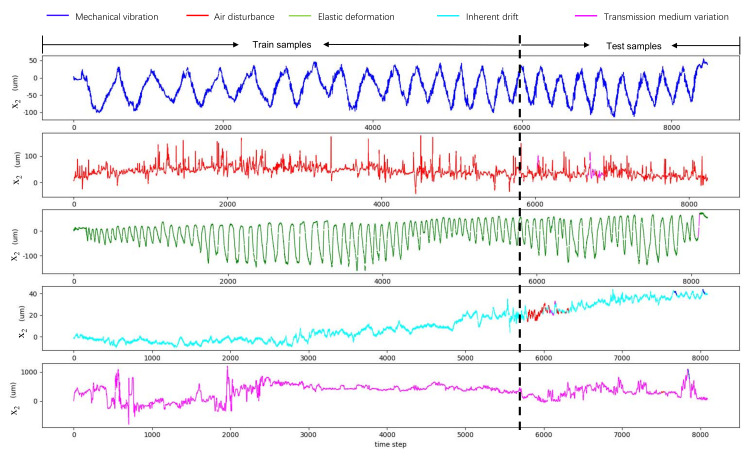
The visualization of the disturbance type classification results of GRU−512. The five rows show the time series under the five different disturbance types, respectively. The dashed line indicates that each time series is divided into a training part and a testing part. The five colors correspond to the five disturbance types. There exists a color inconstancy in the testing part because of the classification errors.

The classification accuracy metric is the percentage of the number of correctly classified samples among all the testing samples. The accuracy and runtime are reported in Table 1. The accuracy of the GRU-512 model was the best, and the runtime satisfied the real-time requirement. The classification results of GRU-512 are visualized in Figure 7, which shows that only a few samples were misclassified.

### 5.3. Beam Pointing Control with Disturbance Source Classification

**Figure 8:**
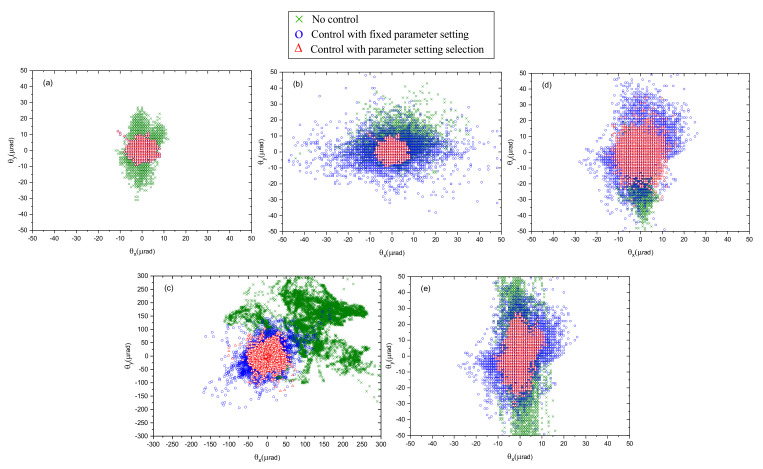
2D beam pointing distributions in the presence of five disturbance types. (**a**) Inherent drift. (**b**) Air disturbance. (**c**) Transmission medium variation. (**d**) Mechanical vibration. (**e**) Elastic deformation. The three colors correspond to different control methods.

Here, we investigate the effectiveness of the beam pointing control system and the influence of using disturbance type classification. Five groups of the target locking experiments were conducted in the disturbances of inherent drifts, air disturbance, transmission medium variations, mechanical motions, and elastic deformations. In each group, three experiments were conducted, which used a disabled controller, a controller with a fixed parameter setting, and a controller with a parameter setting selection. The fixed parameter setting was obtained under the basic disturbance of the inherent drifts. The parameter selection means that beforehand, the five optimal controller parameter settings were tuned under the five disturbance types, respectively, and then, the matched parameter setting was select according to the disturbance type identified by the proposed method when the controller was running. Figure 8 shows the 2D beam pointing $\left(\theta_{x},\theta_{y}\right)$ distributions in the presence of five disturbance types, where $\theta_{x}$ and $\theta_{y}$ respectively represent the beam pointing in the horizontal direction and in the vertical direction. Figure 8 shows that the beam pointing drifts obviously without any control. Beam pointing drift can be reduced using control with fixed parameter setting under some types of disturbance, while beam pointing can not be effectively reduced under some types of disturbance. It is clear that beam pointing drift can be effectively reduced by using the control with the parameter setting selection. Table 2 shows the beam pointing instability under the five disturbance types in the three experiments. A, B, and C represent the experiment of no control, control with a fixed parameter setting, and control with parameter setting selection, respectively. ${∥\theta ∥}_{2}$ is the total RMSE of the horizontal and vertical directions. Beam pointing drift can be effectively reduced with the RMSE under 10 μrad under the four types of disturbance. In addition, the RMSE of beam pointing drift using the proposed method can be reduced to 26.81 μrad from 208.2 μrad under transmission medium variation. From the experiment results, it is shown that the proposed control method can adapt to different types of disturbances. Moreover, the proposed RNN based classification model can identify the disturbance type and then select the best matched control parameter setting automatically.

### 5.4. Discussions

Compared with the kinematic model based on the beam delivery matrix, the kinematic model based on the Jacobian matrix is not only adaptive to the changes of the beam path, but also can be conveniently calibrated by action motions. From the results shown in Section 5.1, it can be seen that the calibration result is adequately accurate. In order to get the kinematic model, it just takes a few seconds using the proposed calibration methods. A classical laser beam control system with fixed control parameters can always perform well when encountering various disturbances. Thus, it is urgent to analyze the characteristics of disturbances and classify the types. To observe the beam pointing drifts under different types of disturbances, we captured the beam pointing drift data under five types of disturbances, which were generated by a transparent box. From the beam pointing drift data, a temporal signal can be obtained by the type of disturbance. As the recurrent neural network is good at time series classification, we propose a disturbance classification method based on the RNN. From the classification results in Section 5.2, the GRU network with 512 hidden neuron numbers has the highest accuracy, and its runtime is 10.1ms, which can satisfy the real-time requirement. Then, we chose the GRU-512 classification model for the laser beam control system. Five groups of target locking experiments were conducted in the mentioned disturbances. The experiments of no control, control with a fixed parameter setting, and control with parameter setting selection were carried out to observe the control performance. Compared with the classical control method with a fixed parameter setting, the RMSE of beam pointing drift can be reduced by 87.12% by the proposed control method. From the experimental results in Section 5.3, the control method with disturbance classification adapts to different types of disturbances. However, some issues should be considered in future work. First, in a practical sense, laser beam control will not experience these five listed disturbances alone. More disturbances need to be studied in future work, so the beam stabilization pointing system can be more universal under various disturbances.Secondly, the proposed beam pointing stabilization methods mainly deal with disturbances under 10Hz because of the limited sampling frequency and hardware bandwidth, so it is necessary to build a high-bandwidth control system with high real-time performance when the frequency of the pulsed laser or disturbances is high. Finally, we will study reinforcement learning by combining classification with parameter adjustment to optimize the control parameters in real-time control.

## 6. Conclusions

In this paper, a beam pointing control system is developed with two FSMs and two PSDs, which can regulate both the position and angle of a laser beam. The kinematic model of the optical path is analyzed by the beam delivery matrix. A Jacobian matrix feedback controller is proposed, which is adaptive to the optical path using the active motion based self-calibration. To realize the intelligent identification of the disturbance type, a generic data-driven disturbance classification method is proposed by using an RNN. The trained RNN model can classify the disturbance type in real time, and the controller’s parameter setting can be better selected. As the experimental results show, beam pointing drift can be effectively reduced with the RMSE under 10 μrad under the four types of disturbances. In addition, the RMSE of beam pointing drift using the proposed method can be reduced to 26.81 rad from 208.2 rad under transmission medium variation. Thanks to the classification of the disturbance type, the control accuracy can be improved when facing various disturbance types.

## Figures and Tables

**Table 1:** Accuracy and training time of the model.

Model	Neuron Number	Accuracy (%)	Time (ms)
GRU	64	92.7	4.2
GRU	128	92.5	4.7
GRU	256	93.9	4.8
GRU	512	94.9	10.1
LSTM	64	92.1	4.4
LSTM	128	91.6	4.5
LSTM	256	91.6	5.0
LSTM	512	91.8	11.3

**Table 2:** Beam pointing instability under five disturbance types (RMSE).

Disturbance Type	Experiment	${∥\theta ∥}_{2}$	$\theta_{x}\left(\mu \mathrm{rad}\right)$	$\theta_{y}\left(\mu \mathrm{rad}\right)$
Inherent drift	A	21.41	2.75	21.23
	B	3.28	2.12	2.43
	C	3.28	2.12	2.43
Air disturbance	A	11.61	7.46	8.89
	B	11.94	10.03	6.47
	C	3.28	2.12	2.43
Transmission medium variation	A	208.20	142.22	152.05
	B	46.46	29.44	35.94
	C	26.81	16.05	21.47
Mechanical vibration	A	19.04	2.60	18.86
	B	21.30	13.01	16.86
	C	7.58	4.34	6.21
Elastic deformation	A	33.56	4.42	33.27
	B	15.15	7.39	13.23
	C	8.71	3.77	7.85
